# Thalamic medial dorsal nucleus atrophy in medial temporal lobe epilepsy: A VBM meta-analysis^☆^

**DOI:** 10.1016/j.nicl.2012.11.004

**Published:** 2012-11-16

**Authors:** Daniel S. Barron, P. Mickle Fox, Angela R. Laird, Jennifer L. Robinson, Peter T. Fox

**Affiliations:** a Research Imaging Institute, UT Health Science Center, San Antonio, TX, USA; b Scott & White, Temple, TX, USA; c Florida International University, Miami, FL, USA; d South Texas Veteran's Health Care System, San Antonio, TX, USA

**Keywords:** Hippocampal sclerosis, Cortical localization, Volumetric MRI, All epilepsy/seizures

## Abstract

**Purpose:**

Medial temporal lobe epilepsy (MTLE) is associated with MTLE network pathology within and beyond the hippocampus. The purpose of this meta-analysis was to identify consistent MTLE structural change to guide subsequent targeted analyses of these areas.

**Methods:**

We performed an anatomic likelihood estimation (ALE) meta-analysis of 22 whole-brain voxel-based morphometry experiments from 11 published studies. We grouped these experiments in three ways. We then constructed a meta-analytic connectivity model (MACM) for regions of consistent MTLE structural change as reported by the ALE analysis.

**Key findings:**

ALE reported spatially consistent structural change across VBM studies only in the epileptogenic hippocampus and the bilateral thalamus; within the thalamus, the medial dorsal nucleus of the thalamus (MDN thalamus) represented the greatest convergence (P < 0.05 corrected for multiple comparisons). The subsequent MACM for the hippocampus and ipsilateral MDN thalamus demonstrated that the hippocampus and ipsilateral MDN thalamus functionally co-activate and are nodes within the same network, suggesting that MDN thalamic damage could result from MTLE network excitotoxicity.

**Significance:**

Notwithstanding our large sample of studies, these findings are more restrictive than previous reports and demonstrate the utility of our inclusion filters and of recently modified meta-analytic methods in approximating clinical relevance. Thalamic pathology is commonly observed in animal and human studies, suggesting it could be a clinically useful indicator. Thalamus-specific research as a clinical marker awaits further investigation.

## Highlights

► Meta-analyzed 11 studies of pathology in medial temporal lobe epilepsy (MTLE). ► The bilateral thalamus is the most consistent extra-hippocampal pathology. ► Within the thalamus, the medial dorsal nucleus (MDN) was the most significant. ► We suggest the MDN thalamus as a target for future studies in MTLE. ► We recommend these modified methods for future VBM meta-analyses.

## 1 Introduction

Medial temporal lobe epilepsy (MTLE) is the most common epilepsy referred to surgical centers and the most common localization-related epilepsy in adults (Téllez-Zenteno and Hernández-Ronquillo, 2012). Hippocampal sclerosis (HS) is the most common pathology in MTLE (also MTLE-HS) and, by diagnostic definition, is the primary site of seizure onset (Wieser and ILAE Commission on Neurosurgery of Epilepsy, 2004). When it is the sole pathology observed, HS is a predictor of positive surgical outcome (Engel, 2008). Conversely, additional extra-hippocampal pathology is a predictor of poor surgical outcome (Thom et al., 2010). Group-averaged morphometric techniques, however, demonstrate that areas of subtle extra-hippocampal pathology often go undetected on visual inspection of individual subjects. These morphometric techniques have also correlated subtle extra-hippocampal pathology with poor surgical outcome (Thom et al., 2010). If an area of consistent extra-hippocampal pathology could be identified across group-averaged studies of MTLE-HS patients, this could inform a targeted, quantitative analysis on the single-subject level. The purpose of this paper is to determine where extra-hippocampal pathology is most consistent across group-averaged studies of MTLE-HS patients.

Voxel-based morphometry (VBM) is a group-averaged morphometric technique used to survey the brain for subtle structural change not observed on visual analysis (Ashburner and Friston, 2000). Many studies have used VBM to localize MTLE-related structural change in both whole-brain and region-specific analyses. VBM studies in MTLE patients constitute a large body of quantitative literature that reports many areas of focal structural change throughout the brain. While the results of voxel-based techniques are not as intuitive as other modalities (e.g. cortical thinning) and criticisms of VBM methods have been reported, the extensive VBM literature permits a robust meta-analysis with a high level of statistical rigor. Furthermore, meta-analytic methods that take advantage of results reported in a standardized, coordinate space are particularly well suited for VBM meta-analysis. Because similar meta-analytic methods do not exist for other morphometric modalities, VBM literature presents a unique opportunity to localize consistent structural change across a large number of independent studies.

Coordinate-based meta-analytic methods are a rapidly evolving field. Anatomic likelihood estimation (ALE) is a well-validated and frequently used coordinate-based meta-analytic technique. At the time of writing, the Eickhoff et al. (2012) ALE algorithm is the latest refinement of the ALE algorithm (see online supplementary materials) and offers unprecedented rigor in identifying which regions and pathways demonstrate the most consistent structural change in MTLE (Eickhoff et al., 2012). Here, we used the Eickhoff et al. (2012) ALE algorithm to quantify consistent structural change from 22 whole-brain VBM experiments representing 519 MTLE patients with unilateral HS. We then used meta-analytic connectivity modeling (MACM) (Robinson et al., 2012) to determine hippocampal co-activation patterns and to evaluate whether areas reported in the VBM meta-analysis could function as nodes in the MTLE epileptogenic network.

## 2 Materials and methods

### 2.1 Paper inclusion filters

We adopted both standard and MTLE-specific paper retrieval filters in selecting papers for inclusion in our meta-analysis (Table 1). (Fox et al., 2005a) The main inclusion criterion was that all studies be whole-brain VBM analyses. We chose an additional criterion for data non-redundancy to avoid within-lab and sample-specific bias. Paper retrieval included an internet and a bibliographic search of relevant retrieved papers.

### 2.2 Internet search

A pubmed.gov search cuing [(“Temporal Lobe Epilepsy” OR TLE) AND (voxel OR “voxel-based morphometry” OR VBM)] returned 164 papers (2.Apr.2012). A breakdown of papers excluded per filter from this pubmed.gov search is found in Table 1. Upon request, Pail et al. (2010) supplied previously unreported VBM foci for inclusion in our meta-analysis.

An OVID search using the same search function with limits to “abstracts,” “English language,” and “all adult (19 plus years)” returned 235 papers (2.Apr.2012). No additional papers were added from this search.

### 2.3 Bibliographic search

All bibliographies of papers that passed our filters were reviewed to find additional papers. No additional papers were added from this search. Eleven papers contained experiments that passed our inclusion filters (see Table 2) (Keller et al., 2002; Bernasconi et al., 2004; Bonilha et al., 2004; McMillan et al., 2004; Mueller et al., 2006; Bouilleret et al., 2008; Pell et al., 2008; Riederer et al., 2008; Pail et al., 2010; Santana et al., 2010; Tae et al., 2010). Our ALE meta-analysis (described below) included 22 experimental contrasts from these papers reporting 300 total foci representing 519 MTLE patients. The majority of studies reported foci in MNI space; those reported in Taiairach and Torneux space were transformed to MNI space via the icbm2tal transform (Lancaster et al., 2007).

### 2.4 Anatomic likelihood estimation (ALE) meta-analysis procedure

Spatial concordance among the reported VBM foci was computed with the modified anatomic likelihood estimation (ALE) algorithm (Eickhoff et al., 2012). As described in previous methods papers (Eickhoff et al., 2009; Turkeltaub et al., 2012), to reflect the spatial uncertainty of reported VBM foci, ALE treats each VBM focus as a Gaussian probability distribution. Gaussian distributions are pooled voxel-wise first within experimental contrasts, then across contrasts within a group to create a whole-brain ALE map. Within this whole-brain ALE map, each voxel is assigned a unique ALE value that represents the likelihood of experimental effects (e.g. gray matter structural change) in that voxel. To reflect the random spatial association between different experiments, ALE maps are tested against a null distribution and are thresholded to a user-specified statistical value. Voxels that pass this statistical threshold are reported as ALE clusters of significant meta-analytic convergence. Recent improvements to the ALE algorithm include: statistical limitations on the effects individual foci can have across experiments and groups (Turkeltaub et al., 2012); an analytic, data-driven approach in creating the null-distribution used in statistical inference (previously an empirical permutation process) (Eickhoff et al., 2012); and a Monte-Carlo based approach to permit more accurate cluster-level inference (Eickhoff et al., 2012). Therefore, as used in our analysis, the Eickhoff et al. (2012) algorithm represents the latest in coordinate-based meta-analysis. All ALE analyses were carried out in GingerALE 2.2, (brainmap.org/ale/index.html) using the Cluster-Level threshold (P < 0.05) with a false discovery rate of (pN, P < 0.05).

We analyzed the 300 VBM foci in 3 groups. To analyze convergence between hemispheres across groups, we rectified L-MTLE VBM foci to the opposite hemisphere by changing the sign of the x-coordinate and concatenating rectified L-MTLE with reported R-MTLE VBM foci. This produced one rectified L + R-MTLE group (Rct). We analyzed convergence between groups across hemispheres in separate R-MTLE (R) and L-MTLE (L) groups.

### 2.5 MACM procedure

Meta-analytic connectivity modeling (MACM) is a meta-analytic technique that investigates whole-brain coactivation patterns for a user-specified region of interest (ROI) (Robinson et al., 2010; Robinson et al., 2012). BrainMap is a neuroimaging database reporting whole-brain functional activation patterns in healthy subjects (Fox et al., 2005b). To determine hippocampal connectivity patterns, we used L and R hippocampal ROIs defined by the Talairach Daemon (Lancaster et al., 2000) and searched the BrainMap database for studies reporting activations within these ROIs (search performed 28.April.2012). We only included retrieved studies reporting results in healthy controls. We integrated whole brain activation patterns from these studies using the ALE method, yielding a MACM-ALE map of significant co-activations (FDR pN p < 0.05). MACM-ALE maps have been validated with diffusion tensor imaging (DTI) and connectivity atlases (CocoMac) (Robinson et al., 2010) and have been demonstrated to be the meta-analytic equivalent of resting-state functional connectivity maps (Laird et al., 2009; Smith et al., 2009).

## 3 Results

### 3.1 ALE

Results of the 3 ALE analyses are presented in Table 3. Here in text, we report areas of statistically significant meta-analytic convergence that represent > 30% of papers; all reported areas are presented in Supplementary data. For each area we report the location of maximum ALE values (ALEmx) as (x, y, z) coordinates in MNI space.

#### 3.1.1 Rectified L + R-MLTE analysis

The largest area (volume) of consistent gray matter structural change was centered in the epileptogenic hippocampus (weighted center = 32, − 16, − 18; volume = 2112 mm^3^); it encompassed 1 ALEmx in the epileptogenic hippocampus. The second largest area of consistent structural change was in the bilateral thalamus (weighted center = 0, − 17, 9; volume = 1632 mm^3^); it encompassed 2 ALEmx in the contralateral medial dorsal nucleus and the contralateral ventral posterior medial nucleus.

#### 3.1.2 L-MTLE analysis

The largest area of consistent gray matter structural change was in the epileptogenic hippocampus (weighted center = − 31, − 16, − 18; volume = 904 mm^3^); this encompassed 1 ALEmx. The second largest area of consistent structural change was specifically the ipsilateral medial dorsal nucleus of the thalamus (weighted center = 3, − 16, 5; volume = 160 mm^3^); this encompassed 1 ALEmx.

#### 3.1.3 R-MTLE analysis

The largest area of consistent gray matter structural change was found in the epileptogenic hippocampus (weighted center = 34, − 17, − 17; volume = 1184 mm^3^); this encompassed 1 ALEmx. The second area of consistent structural change was found in the contralateral ventral posterior median nucleus of the thalamus (weighted center = − 14, − 17, 7; volume = 200 mm^3^); this encompassed 1 ALEmx.

#### 3.1.4 Conditional probability of thalamus and hippocampus

We computed the conditional probability that each VBM experiment reported structural change (as an x, y, z focus) within each of our reported ALE clusters (Table 4). It should be noted that the conditional probability that each VBM experiment reported *both* author-labeled thalamic *and* hippocampal structural change was 1.0, i.e. thalamic and hippocampal labels were co-reported in 100% of experiments. It should also be noted that our reported conditional probabilities are per paper (i.e., per group) and cannot be assumed to be predictive at the per subject level. Per subject conditional probabilities must be determined from per subject data in future studies.

#### 3.1.5 ALE summary

The three group ALE analyses yielded similar results: significant spatial convergence across studies exists only in the epileptogenic hippocampus and the bilateral thalamus. Of the different thalamic regions, the MDN thalamus represents the greatest consensus. A comparison of these results to results from previously-published coordinate-based meta-analysis methods is found in the supplementary online materials.

### 3.2 MACM

We created two meta-analytic connectivity models (MACM) for separate L and R hippocampal ROIs based on tissue labels from the Talairach-Daemon (Lancaster et al., 2000). The purpose of these analyses was to test whether and where the hippocampus co-activated with the bilateral thalamus and whether these areas could serve as nodes within the MTLE epileptogenic network (described below). Therefore we only report pertinent MACM results from bilateral hippocampus and thalamus.

#### 3.2.1 L. Hipp

**Fig. 1:**
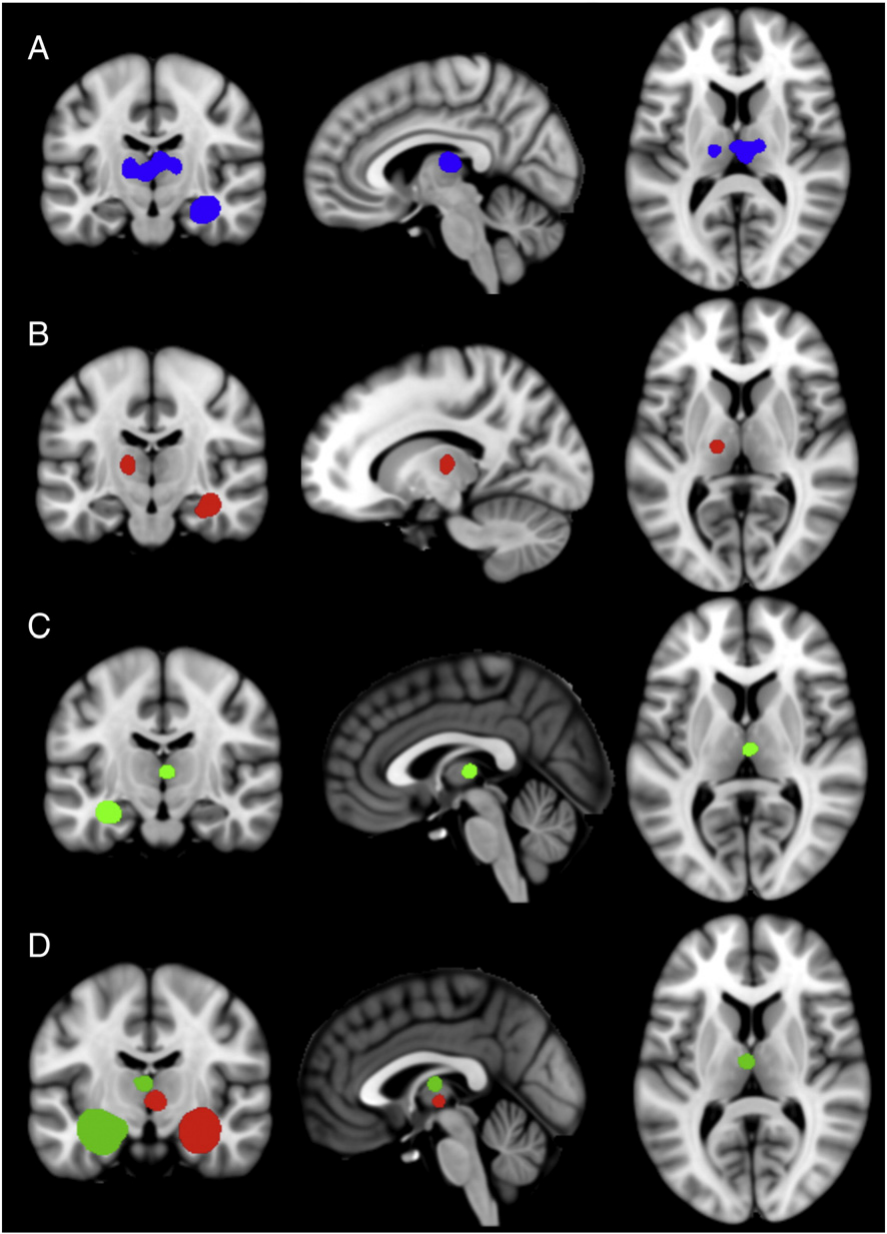
Significant areas of convergence in the VBM-ALE analysis in rectified (A), right medial temporal lobe epilepsy (B), and left medial temporal lobe epilepsy (C) groups. MACM analyses (D) of the left (green) and right (red) hippocampal tissue labels; note co-activation in the medial dorsal nucleus thalamus. All clusters thresholded at P < 0.05, corrected for multiple comparisons. See Supplementary materials for full montages of (A), (B) and (C).

The BrainMap search returned 481 foci from 38 experiments representing 554 subjects. Resultant ALE maps demonstrated strong bilateral hippocampal, ipsilateral MDN, and contralateral ventral anterior nucleus co-activity (Fig. 1-E).

#### 3.2.2 R. Hipp

The BrainMap search returned 402 foci from 28 experiments representing 474 subjects. ALE maps demonstrated strong bilateral hippocampal and ipsilateral MDN co-activity (Fig. 1-E).

#### 3.2.3 MACM summary

These results demonstrate hippocampal functional co-activity with the ipsilateral MDN thalamus and suggest that these areas serve as nodes within the same network. An illustrative diffusion tractography analysis in a healthy subject (n = 1) confirms strong white matter connections between the left MDN thalamus and hippocampus. (Fig 1-F) This preliminary diffusion tractography was created within 3D Slicer (Pieper et al., 2006) and is only intended to illustrate potential pathways of hippocampo-thalamic anatomic connectivity which will be studied in future analyses.

## 4 Discussion

Medial temporal lobe epilepsy (MTLE) is associated with MTLE network pathology within and beyond the hippocampus. The purpose of this meta-analysis was to identify consistent MTLE structural change to guide subsequent targeted analyses of these areas. To this end we performed an anatomic likelihood estimation (ALE) meta-analysis of 22 whole-brain voxel-based morphometry (VBM) experiments grouped in three ways. Only the epileptogenic hippocampus and the bilateral thalamus showed spatially consistent structural change across VBM studies; within the thalamus, the medial dorsal nucleus of the thalamus (MDN thalamus) represented the greatest convergence. We then constructed a meta-analytic connectivity model (MACM) for the hippocampus and ipsilateral MDN thalamus. This demonstrated that the hippocampus and ipsilateral MDN thalamus functionally co-activate and are nodes within the same network, suggesting that MDN thalamic damage could result from MTLE network excitotoxicity.

### 4.1 MTLE connectivity

Because the hippocampus is a central component of the MTLE epileptogenic network, we will discuss its connectivity profile. Specifically, we are interested in the three most notable regions (or nodes) of the MTLE epileptogenic circuit – entorhinal cortex, contralateral hippocampus, and thalamus – and why the MDN thalamus is only area within the MTLE epileptogenic network to demonstrate consistent structural change across VBM studies.

The entorhinal cortex (EC) is the main afferent projection to the hippocampus via the perforant pathway. Entorhinal–hippocampal circuitry has been rigorously studied in animal models as a gatekeeper of information flow between the neocortex and hippocampus and as part of the MTLE epileptogenic network (Lavenex and Amaral, 2000; Bernhardt et al., 2008). Human studies demonstrate that the EC is able to generate spontaneous ictal events and that the EC may have a lower threshold for seizure generation than the hippocampus. Some volumetric studies have demonstrated EC pathology ipsilateral to hippocampal sclerosis (Keller and Roberts, 2008). Studies have also demonstrated that when present, the degree of EC neuropathology is correlated to its involvement in MTLE seizure genesis (Bartolomei et al., 2005). Group studies involving the EC have demonstrated resting state functional magnetic resonance imaging (rs-fMRI) connectivity abnormalities; however, this pathology is inconsistent on the individual patient level and is therefore of limited clinical utility (Bettus et al., 2010).

Hippocampi share direct inter-hemispheric connections via the hippocampal commissure. Animal studies (monkey and rat) demonstrate clear and numerous commissural connections mainly from CA3 to contralateral hippocampus (Spencer et al., 1987). Human studies demonstrate that seizures originating in the epileptogenic hippocampus spread to the contralateral hippocampus both with and without the simultaneous involvement of ipsilateral neocortex; thus operationally validating the importance of the hippocampal commissure in humans (Spencer et al., 1987). While inter-hippocampal seizure propagation time varies across patients and has been inversely correlated to epileptogenic hippocampal damage (CA4), contralateral hippocampal pathology is observed in only a minority of volumetric studies (Spencer et al., 1992; Keller and Roberts, 2008).

Thalamo-hippocampal connectivity is well established in animal models and in human temporal lobe epilepsy patients (Vertes et al., 2007; Guye et al., 2006). Thalamic regions most commonly associated with temporal lobe epilepsy are the pulvinar, the anterior nucleus, and the medial dorsal nucleus. Intracerebral electrophysiology studies in temporal lobe epilepsy patients have detected ictal activity in the medial pulvinar of the thalamus, likely related to its reciprocal connections with the temporal lobe (Rosenberg et al., 2006; Rosenberg et al., 2009). Because none of Rosenberg's patients had *medial* temporal lobe epilepsy and over half (9 of 14) demonstrated multifocal seizure onset outside the medial temporal lobe, this study does not directly apply to MTLE patients but does underline the importance of the thalamus in temporal lobe seizure propagation (Rosenberg et al., 2006). The anterior nucleus of the thalamus projects both to superior frontal and temporal lobe structures, in particular with the subicular portions of the hippocampal formation (Aggleton et al., 2010). Bilateral stimulation of the anterior nucleus effectively reduced seizure activity in a multi-center, double-blind, randomized trial of 110 patients with partial and secondarily generalized seizures (Fisher et al., 2010). Because 66% of these patients had temporal lobe epilepsy, it could be said that the anterior nucleus plays a modulatory role in the epileptogenic circuit. While the medial pulvinar and the anterior nucleus have been associated with temporal lobe epilepsy, they have not been shown to be heavily involved its diagnostic sub-classification, MTLE.

The medial dorsal nucleus of the thalamus has been associated specifically with MTLE. Animal models of MTLE have shown the MDN thalamus is a central contributor to seizure onset and acts as a common hub, synchronizer, and perhaps even regulator of secondary generalization from the MTL to distant neocortical areas (Bertram et al., 2008). Ictal SPECT studies in MTLE patients have shown consistent blood flow increases in ipsilateral medial thalamus and MTL at seizure onset, demonstrating participation of the medial thalamic region in MTLE networks and its potential involvement in seizure initiation (Spencer, 2002). Electrophysiological studies in human MTLE patients confirm increased synchronization between the thalamus and hippocampus during seizure activity and speculate that this thalamo-hippocampal coupling may amplify seizure activity (Guye et al., 2006). Recent diffusion tractography analyses confirm previous tracer studies, describing anatomic connections specifically between the MDN thalamus and the hippocampus (Behrens et al., 2003).

Notably, our meta-analysis demonstrates the most consistent structural change in the MDN thalamus, not in other MTLE network areas. We have further validated hippocampal-MDN connectivity with a meta-analytic connectivity model.

### 4.2 MDN thalamic pathology in MTLE

Thalamic pathology in TLE has much historical precedent (Delasiauve, 1854; Margerison and Corsellis, 1966). Global thalamic volume loss is a common observation and implies that neurons throughout the thalamus are pathologically affected by temporal lobe seizure activity (Deasy et al., 2000). Animal models of kindled MTLE have shown consistent medial dorsal thalamic neuronal loss (Bertram and Scott, 2000). Three-dimensional surface modeling of high-resolution MRI demonstrated strong medial thalamic atrophy in human MTLE (Bernhardt et al., 2012). Our VBM meta-analysis demonstrates that the MDN thalamus is the most significant and consistent extra-hippocampal area of structural change overall.

This raises the question: Why do we detect structural change in the MDN thalamus but not in other areas? One possibility: there is something particularly atrophogenic about thalamohippocampal coupling that causes more consistent, pronounced damage in the MDN thalamus than to other areas. Another possibility: all MTLE network pathways are equally atrophogenic, but because non-thalamic pathways are variable across patients, this causes heterogeneous areas of structural change that are not detected by VBM due to group averaging. Recent efforts to sub-categorize MTLE based on heterogenous hippocampal pathology (Blumcke et al., 2007) could support this possibility by causing preferential involvement of heterogeneous non-thalamic pathways. Along the same lines, sub-categorization of hippocampal pathology could also explain variations in contralateral hippocampal propagation time and order across patients, described above.

### 4.3 Methodological considerations

VBM is a voxel-wise, whole-brain image analysis strategy capable of detecting subtle anatomic structural change that appears normal on conventional MRI (Ashburner and Friston, 2000). VBM is among the most used (if not the most) quantitative volumetric analysis technique (cited in over 3100 peer-reviewed publications, 20.April.2012). A major challenge for anatomic lesion detection is to sufficiently normalize structures to permit meaningful comparison (e.g. disease vs. control) without diluting or abrogating the signal of interest (e.g. gray matter reduction). To the degree these techniques fail, VBM will produce inconsistent false negative and false positive results; as with most statistical tests these errors are mitigated by increased sample size. These challenges contribute to VBM's lack of replication across studies and have been clearly and scholarly reviewed in Keller and Roberts (2008). We invite readers to refer to Keller and Roberts (2008) for further discussion of VBM methodology. Collectively, these challenges reduce the reliability of individual studies and necessitate the integration of several studies to identify areas of reliable brain structural change. Because of the large body of VBM literature, VBM studies can be integrated through meta-analysis thus allowing the most consistent (and most likely to be true positive) results to be identified. This integration can be best accomplished through coordinate-based meta-analysis, as we have done.

Meta-analysis is most generally defined as the post hoc combination of independently performed studies to better estimate a parameter of interest (Fox et al., 2005a). Label-based meta-analysis is a non-statistical form of meta-analysis wherein author-assigned labels from multiple studies are tallied and plotted. Keller and Roberts (2008) performed a label-based meta-analysis of 18 whole-brain and region-specific VBM studies. Similar to our findings, Keller and Roberts (2008) reported the most frequent gray matter structural change in the epileptogenic hippocampus (82.35%) and ipsilateral thalamus (61.11%). Unlike our findings, they report significant structural change in 24 additional brain regions. This divergence is not surprising: a long-established critique of label-based meta-analyses is that author-assigned labels are spatially imprecise and can produce misleading results if pooled across studies (Laird et al., 2005). While Keller and Roberts (2008) provided a balanced introduction of VBM techniques to the epilepsy community (an explicit goal of the paper), their label-based review had many methodological shortcomings: tallied, spatially imprecise labels were used to integrate studies; a heterogeneous sampling of region-based and whole-brain VBM analyses were combined into one data pool; and multiple studies representing the same patient sampling and laboratory introduced redundancy to the analysis, thereby biasing the reported effects. It is not unexpected that this study failed to find a clear or a reliable consensus of MTLE structural change.

Coordinate-based meta-analysis offers a statistically rigorous, spatially precise alternative to label-based meta-analysis. In addition, coordinate-based meta-analysis is also a more feasible alternative to image-based meta-analysis which requires that original statistical parametric maps be gathered from each published study. As one might expect, statistical parametric maps are often not available for large-scale, multi-laboratory meta-analysis, as they were not in our case. Coordinate-based meta-analysis makes large-scale, multi-laboratory meta-analysis possible by taking advantage of information already available in the published literature.

Anatomic Likelihood Estimation (ALE) is the most-used form of coordinate-based meta-analysis. Recently, Li et al. (2012) performed an ALE meta-analysis of 6 whole-brain VBM studies of MTLE patients. Li et al. (2012) used the Eickhoff et al. (2009) ALE algorithm, which was the latest ALE algorithm at the time of publishing. Li et al. (2012) reported a more refined and more restrictive spatial convergence (P < 0.05, corrected for multiple comparisons) than Keller and Roberts (2008) that included the bilateral thalamus and ipsilateral MTL structures such as the hippocampus, parahippocampus, insula. While Li et al. (2012) was able to localize consistent brain structural change better than Keller and Roberts (2008), Li et al. (2012) did not provide a clear consensus of MTLE structural change.

Our study takes advantage of significant improvements in meta-analytic methods, to good effect. First, we applied inclusion filters for whole-brain analyses (no regional studies) and data non-redundancy. Second, we analyzed 22 experiments from 11 studies in separate L-MTLE and R-MTLE groups and as a rectified group to increase statistical power across L and R groups. Third, we used the recently released Eickhoff et al. (2012) ALE algorithm. This algorithm not only incorporates previously unavailable modifications to statistical effect size,(Turkeltaub et al., 2012) but also uses a novel, data-driven approach to create the null-distribution used in statistical inference (previously an empirical permutation process) and a Monte-Carlo based approach to permit more accurate cluster-level inference (Eickhoff et al., 2012). These improvements to the meta-analytic process represents the latest in meta-analytic methods and provide significant improvements in precision as demonstrated in the supplementary online materials. Because our study yielded distilled, powerful results, we recommend future clinically oriented ALE meta-analyses utilize these methods.

### 4.4 Clinical purpose and dividends

The purpose of this meta-analysis was to identify spatially consistent regions of MTLE structural change to guide future clinical studies. Because VBM represents the largest body of quantitative structural analyses in MTLE, we applied the most powerful meta-analytic methods to existing MTLE VBM data. Our meta-analysis reports strong, consistent MTLE-related structural change only within the epileptogenic hippocampus and bilateral thalami. While the possibility of additional neocortical structural change remains, we have no evidence that VBM (or any other technique) can detect it in a spatially consistent manner. This may be a methodological shortcoming of VBM (i.e. if VBM were more sensitive, it could consistently detect additional structural change) however, the fact remains that VBM detects spatially consistent thalamic structural change.

Thalamic pathology in MTLE is very well supported by the animal-model literature and is represented in the clinical literature, although previously it has not been identified with such clarity. We suggest future group-averaged studies target the thalamus, specifically the MDN thalamus. Targeted analysis of the thalamus could subsequently bear prospective clinical dividends on the per-patient level by supplementing the pre-surgical evaluation, mitigating surgical failure by identifying patients that are less likely to benefit from surgery, and informing new therapeutic interventions.

## 5 Conclusion

In unilateral MTLE, we found consistent gray matter structural change only within the epileptogenic hippocampus and bilateral thalami. The MDN thalamus represented the most significant thalamic structural change — nearly equal to the epileptogenic hippocampus. Notwithstanding our large sample of studies, these findings are more restrictive than previous reports and demonstrate the utility of our inclusion filters and of recently modified meta-analytic methods in approximating clinical relevance. Thalamic pathology is commonly observed in animal and human studies, suggesting it could be a clinically useful indicator. Thalamus-specific research as a clinical marker awaits further investigation.

## Disclosure of conflicts of interest

The authors have no conflicts of interest to report.

We confirm that we have read the Journal's position on issues involved in ethical publication and affirm that this report is consistent with those guidelines.

## Figures and Tables

**Table 1:** Inclusion filters and papers eliminated by each filter for the pubmed.gov search.

Filter	Description	Justification	Number of papers	Number eliminated
Internet search	[(“Temporal Lobe Epilepsy” OR TLE) AND (voxel OR “voxel-based morphometry” OR VBM)]		164	
Relevance	Published in English-language, peer-reviewed journals; reported^a^ x–y–z foci from whole-brain VBM (modulated or unmodulated) analyses in MNI or T&T space	Standard relevance criteria (Fox et al., 2005a,b)	164	142
MTLE-HS diagnosis	Pre-surgical adult MTLE with unilateral HS verified with EEG, structural MRI, and/or post-surgical histology	The majority of papers reported this diagnostic criteria	22	1
Experimental Contrast	Analyses of gray matter reduction in separate R and L-MTLE populations compared to healthy controls, unregressed for other variables. (P < 0.05, uncorr. or more stringent)	Excluded analyses included white matter reduction, R vs L-MTLE, and regressions for aura, disease duration, etc.	21	8
Non-redundancy	Only one contrast per paper per group per patient population	Some groups reported multiple analyses of a patient population within and across papers	13	2
TOTAL			11	

TLE = temporal lobe epilepsy; VBM = voxel-based morphometry; MNI = Montreal Neurological Institute; T&T = Talairach and Tournoux; MTLE = medial temporal lobe epilepsy; HS = hippocampal sclerosis; R = right; L = left.

a Upon request, Pell et al. (2008) supplied previously unreported foci.

**Table 2:** Overview of included studies.

Paper	n (L-MTLE)	n (R-MTLE)	n (controls)	Age^a^	L-MTLE foci^b^	R-MTLE foci^b^	VBM analysis	Smoothing kernel	Significance level^c^	Diagnosis of TLE according to^d^	Unilateral HS?	Patient type	Software: template
Bernasconi et al., 2004	45	40	47	35 ± 10	26	13	UM	5 mm FWHM	P (corr.) < 0.05	video-EEG monitoring + HS	Yes	Unspecified	INSECT: MNI
Bonilha et al., 2004	22	21	49	32 ± 8	20	14	UM	Unspecified	P (uncor.) < .05	89' ILAE Criteria	Yes	Outpatients	SPM2: MNI
Bouilleret et al., 2008	12	0	30	36 ± 10	12	0	M	12 mm FWHM	P (uncorr.) < 0.001	EEG-monitor; structural MRI	Yes	Unspecified	SPM2:T&T
Keller et al., 2002	58	58	58	34	14	13	UM	10 mm FWHM	P (corr.) < 0.05	Scalp + Invasive EEG	Unspecified	Pre-surgical	SPM99:T&T
McMillan et al., 2004	13	12	62	32.2 ± 11	7	15	M	12 mm FWHM	P (corr.) < 0.05	video-EEG monitoring + HS	Yes	Pre-Surgical	SPM2: MNI
Mueller et al., 2006	0	26	30	35.6 ± 9.7	0	33 (R + Rct L)	M + UM	8 mm FWHM	P (corr.) < 0.05	Video-EEG Telemetry	Yes	Pre-surgical	SPM2: MNI
Pail et al., 2010	20	20	40	39.5 ± 9.7	6	11	M	Unspecified	P (corr.) < 0.001, 100 voxels	89' ILAE Criteria	Yes	Unspecified	SPM2: MNI
Pell et al., 2008	19	0	115	39.3 ± 12	18	0	M	6 mm FWHM	P (corr.) < 0.05	Institutional Criteria	Yes	Pre-surgical	SPM2: MNI
Riederer et al., 2008	9	13	12	38 ± 11	10	3	M	4 mm FWHM	P (uncorr.) < 0.001	Video-EEG + HS	Yes	Pre-surgical	SPM2: MNI
Santana et al., 2010	59	41	30	37.29	16	10	M	8 mm FWHM	P (uncorr.) < 0.001	Video-EEG Telemetry	Yes	Pre-surgical	SPM5: MNI
Tae et al., 2010	16	15	47	R: 29 L: 33	28	31	M + UM	8 mm FWHM	P (corr.) < 0.05	EEG monitoring + HS	Yes	Pre-surgical	SPM2: MNI
Total	273	246	520		157	143							

L-MTLE = left medial temporal lobe epilepsy; R-MTLE = right medial temporal lobe epilepsy; MNI = Montreal Neuroimaging Institute; FWHM = Full Width at Half Maximum; Corr. = corrected; Uncorr. = Uncorrected; HS = hippocampal sclerosis; UM = unmodified; M = Modified; Rct = Rectified (consult methods for further explanation); T&T = Talairach and Tournoux.

a Of MTLE patients. Presented in mean ± SD(range) years.

b Foci listed report gray matter reduction only.

c Significance levels reported on whole-brain level.

d Terminology used in the original articles.

**Table 3:** Anatomic likelihood estimation (ALE) summary for Rct, L-MTLE, and R-MTLE analyses.

	Cluster #	Label		Volume (mm^3^)	Weighted center (x, y, z)			ALE Mx (× 10^2^)	Total foci	% Rep
Rct	1	I	Hippocampus	2112	32	− 16	− 18	7.8	23	77%
	2	B	Thalamus	1632	0	− 17	9	3.7	18	55%
			– Med Dorsal Nuc					3.7		
			– Vent Post Med Nuc					3.4		
	3	I	Limbic Lobe.BA 30	176	25	− 38	1	3.2	3	14%
LTLE	1	I	Hippocampus	904	− 31	− 16	− 18	4.8	7	64%
	2	I	Thalamus. Med Dorsal Nuc	160	3	− 16	5	2.5	3	27%
RTLE	1	I	Hippocampus	1184	34	− 17	− 17	3.5	12	82%
	2	C	Thalamus. Vent Post Med Nuc	200	− 14	− 17	7	2.5	2	18%

Rct = Rectified (R-MTLE foci + L-MTLE foci rectified to R hemisphere); L = left medial temporal lobe epilepsy; R = right medial temporal lobe epilepsy; ALEmx = maximum ALE score for listed cluster; B = bilateral; I = ipsilateral to epileptogenic hippocampus; C = contralateral to epileptogenic hippocampus; % Rep = number of studies contributing VBM foci to ALE cluster/number of total studies. All (x, y, z) foci are reported in Montreal Neuroimaging Institute (MNI) space. All tissue labels are derived from the Talairach Daemon.

**Table 4:** Conditional probability of observing thalamic and hippocampal structural changes. We tabulated whether each VBM experiment reported structural change (as an x, y, z focus) within each of our reported ALE clusters. It should be noted that the conditional probability that each VBM experiment reported both author-labeled thalamic and hippocampal structural change was 1.0, i.e. thalamic and hippocampal labels were co-reported in 100% of experiments.

	Number of foci	Probability (P)
*Rct*		
Report Hipp Struct Change	Hipp = 7	P(Hipp) = 0.32
Report Thal Struct Change	Thal = 2	P(Thal) = 0.09
Report Both	Hipp&Thal = 10	P(Hipp&Thal) = 0.45
Report Hipp, Thal, or Hipp&Thal	Total = 19	P(Hipp OR Thal OR Hipp&Thal) = 0.86

*L-MTLE*		
Report Hipp Struct Change	Hipp = 6	P(Hipp) = 0.55
Report Thal Struct Change	Thal = 2	P(Thal) = 0.18
Report Both	Hipp&Thal = 1	P(Hipp&Thal) = 0.09
Report Hipp, Thal, or Hipp&Thal	Total = 9	P(Hipp OR Thal OR Hipp&Thal) = 0.82

*R-MTLE*		
Report Hipp Struct Change	Hipp = 8	P(Hipp) = 0.36
Report Thal Struct Change	Thal = 1	P(Thal) = 0.05
Report Both	Hipp&Thal = 1	P(Hipp&Thal) = 0.05
Report Hipp, Thal, or Hipp&Thal	Total = 10	P(Hipp OR Thal OR Hipp&Thal) = 0.45

Rct = rectified (R-MTLE foci + L-MTLE foci rectified to R hemisphere); L = left medial temporal lobe epilepsy; R = right medial temporal lobe epilepsy; Struct = Structural; Hipp = Hippocampus; Thal = Thalamus.
