# Supplementary material for: Thalamic medial dorsal nucleus atrophy in medial temporal lobe epilepsy: A VBM meta-analysis
Source: Neuroimage Clin. 2012 Nov 16;2:25–32. doi: 10.1016/j.nicl.2012.11.004 (PMC3777772; doi:10.1016/j.nicl.2012.11.004)

**Supporting Online Material:**

*Demonstration of the Eickhoff (2009) and Eickhoff (2012) ALE algorithms*

As discussed in the main text, the Eickhoff (2012) ALE algorithm has significant methodological improvements over the previous Eickhoff (2009) algorithm. Stepwise improvements to the Eickhoff (2009) algorithm have included minimization of within-experiment and within-group statistical bias (Turkeltaub, 2012); and substitution of a permutation algorithm with a faster, more rigorous analytic solution for the null-distribution and modification of the multiple comparisons procedure. (Eickhoff, 2012) Each improvement has provided a more precise tool for localizing spatially-consistent experimental effects across studies.

In the Supporting Table below, we present a comparison of the Eickhoff (2009) and Eickhoff (2012) algorithms. Here, we meta-analyze the rectified (R + L rectified to R) group from main text, including 22 whole-brain voxel-based morphometry experiments of gray matter reduction. Both analyses had similar statistical (P<0.05 corrected) and volume (100mm^3^) thresholds.

The Eickhoff (2012) algorithm only reports the most significant regions of spatial convergence across studies, eliminating clusters 5-14 reported with the Eickhoff (2009) algorithm. Clusters 5-14 were represented by few of the included studies (only 9-18%) and were likely experimental noise, not representative of true pathology in the population at-large. Also of note is the absence of the caudate cluster (cluster 3) in the Eickhoff (2012) report. Even though this cluster was represented by 27% of experiments included in analysis, it was not reported as statistically significant in the Eickhoff (2012) algorithm. Upon further investigation, we discovered that this cluster was created by 11 VBM foci, 5 of which were reported by McMillan (2004). This is known as within-experiment bias and causes false-positive statistical significance. Because within-experiment bias was corrected for in the Turkeltaub (2012) ALE algorithm (implemented in the Eickhoff (2012)), the caudate cluster was not reported as statistically significant in the Eickhoff (2012) analysis. To date, the Eickhoff (2012) represents the latest in meta-analytic methods.

**References:**

Eickhoff SB, Bzdok D, Laird AR, Kurth F, Fox PT. (2012) Activation likelihood estimation meta-analysis revisited. *NeuroImage* 59:2349-2361.

Eickhoff SB, Laird AR, Grefkes C, Wang LE, Zilles K, Fox PT. (2009) Coordinate-based activation likelihood estimation meta-analysis of neuroimaging data: A random-effects approach based on empirical estimates of spatial uncertainty. *Human brain mapping* 30:2907-2926.

McMillan AB, Hermann BP, Johnson SC, Hansen RR, Seidenberg M, Meyerand ME. (2004) Voxel-based morphometry of unilateral temporal lobe epilepsy reveals abnormalities in cerebral white matter. *NeuroImage* 23:167-174.

Turkeltaub PE, Eickhoff SB, Laird AR, Fox M, Wiener M, Fox P. (2012) Minimizing within-experiment and within-group effects in activation likelihood estimation meta-analyses. *Human brain mapping* 33:1-13.

**Supporting Table.** Comparison of reports from Eickhoff (2009) and Eickhoff (2012) algorithm. Both analyzed the Rectified MTLE group, described in the main text using similar statistical (P<0.05 corrected) and volume (100mm^3^) thresholds. Consistent clusters across reports are highlighted in blue.

|  |  | Eickhoff (2009) ALE Algorithm | | | | | | | | Eickhoff (2012) ALE Algorithm | | | | | | | |
| --- | --- | --- | --- | --- | --- | --- | --- | --- | --- | --- | --- | --- | --- | --- | --- | --- | --- |
|  | | Cluster # | Volume (mm^3^) | Weighted Center (x,y,z) | | | ALE Mx (x10^2^) | Total Foci | % Rep | Cluster # | Volume (mm^3^) | Weighted Center (x,y,z) | | | ALE Mx (x10^2^) | Total Foci | % Rep |
| Label | |  |  |  |  |  |  |  |  |  |  |  |  |  |  |  |  |
| B | Thalamus | 1 | 8536 | 1 | -17 | 9 | 3.7 | 31 | 77% | 2 | 1632 | 0 | -17 | 9 | 3.7 | 18 | 55% |
|  | Med Dorsal Nuc |  |  |  |  |  | 3.7 |  |  |  |  |  |  |  | 3.7 |  |  |
|  | Vent Post Med Nuc |  |  |  |  |  | 3.4 |  |  |  |  |  |  |  | 3.4 |  |  |
| I | Hippocampus | 2 | 5984 | 32 | -16 | -18 | 7.8 | 33 | 77% | 1 | 2112 | 32 | -16 | -18 | 7.8 | 23 | 77% |
| I | Caudate | 3 | 1640 | 10 | 8 | 11 | 1.7 | 11 | 27% |  |  |  |  |  |  |  |  |
|  | Caudate Body |  |  |  |  |  | 1.5 |  |  |  |  |  |  |  |  |  |  |
| I | Limbic Lobe.BA 30 | 4 | 1296 | 25 | -38 | 1 | 3.2 | 5 | 23% | 3 | 176 | 25 | -38 | 1 | 3.2 | 3 | 14% |
| C | Cerebellum.Pyramis | 5 | 624 | -24 | -81 | -34 | 1.8 | 4 | 18% |  |  |  |  |  |  |  |  |
| C | Paracentral Lobule.BA 31 | 6 | 456 | -1 | -10 | 47 | 1.5 | 3 | 14% |  |  |  |  |  |  |  |  |
| I | Cerebellar Tonsil | 7 | 320 | 32 | -61 | -35 | 1.7 | 2 | 9% |  |  |  |  |  |  |  |  |
| I | Subcallosal Gyrus.BA 25 | 8 | 320 | 11 | 24 | -23 | 1.8 | 2 | 9% |  |  |  |  |  |  |  |  |
| I | Limbic Lobe.BA 36 | 9 | 312 | 35 | -37 | -17 | 1.7 | 2 | 9% |  |  |  |  |  |  |  |  |
| C | Cerebellar Tonsil | 10 | 288 | -31 | -61 | -35 | 1.6 | 2 | 9% |  |  |  |  |  |  |  |  |
| I | Cerebellum.Culmen | 11 | 272 | 26 | -34 | -31 | 1.8 | 2 | 9% |  |  |  |  |  |  |  |  |
| C | Cingulate Gyrus.BA 32 | 12 | 256 | 2 | 30 | 29 | 1.8 | 2 | 9% |  |  |  |  |  |  |  |  |
| I | Putamen | 13 | 240 | 36 | -6 | -5 | 1.4 | 2 | 9% |  |  |  |  |  |  |  |  |
| I | Cerebellum.Pyramis | 14 | 224 | 24 | -80 | -30 | 1.5 | 2 | 9% |  |  |  |  |  |  |  |  |
| C | Precentral Gyrus.BA 4 | 15 | 216 | -55 | -9 | 35 | 1.5 | 2 | 9% |  |  |  |  |  |  |  |  |
| I | Med Frontal Gyrus.BA 6 | 16 | 216 | 4 | -19 | 61 | 1.4 | 2 | 9% |  |  |  |  |  |  |  |  |
| I | Sup Temporal Gyrus.BA 22 | 17 | 200 | 69 | -41 | 17 | 1.8 | 1 | 5% |  |  |  |  |  |  |  |  |
| C | MidFrontal Gyrus.BA 6 | 18 | 184 | -26 | 1 | 64 | 1.5 | 2 | 9% |  |  |  |  |  |  |  |  |
| C | MedFrontal Gyrus.BA 10 | 19 | 176 | 0 | 62 | 4 | 1.4 | 2 | 9% |  |  |  |  |  |  |  |  |
| I | Med Frontal Gyrus.BA 6 | 20 | 160 | 3 | 8 | 51 | 1.4 | 2 | 9% |  |  |  |  |  |  |  |  |
| I | Sup Frontal Gyrus.BA 6 | 21 | 128 | 18 | 31 | 55 | 1.2 | 1 | 5% |  |  |  |  |  |  |  |  |
| I | Inf Temporal Gyrus.BA 20 | 22 | 112 | 69 | -28 | -21 | 1.3 | 1 | 5% |  |  |  |  |  |  |  |  |

Rct=Rectified (R-MTLE foci + L-MTLE foci rectified to R hemisphere); L=left medial temporal lobe epilepsy; R=right medial temporal lobe epilepsy; ALEmx=maximum ALE score for listed cluster; B=bilateral; I=ipsilateral to epileptogenic hippocampus; C=contralateral to epileptogenic hippocampus; % Rep=number of studies contributing VBM foci to ALE cluster/number of total studies.

^a^ All (x,y,z) foci are reported in Montreal Neuroimaging Institute (MNI) space.

^b^ All tissue labels are derived from the Talairach Daemon.

**Supporting Figure 1:**


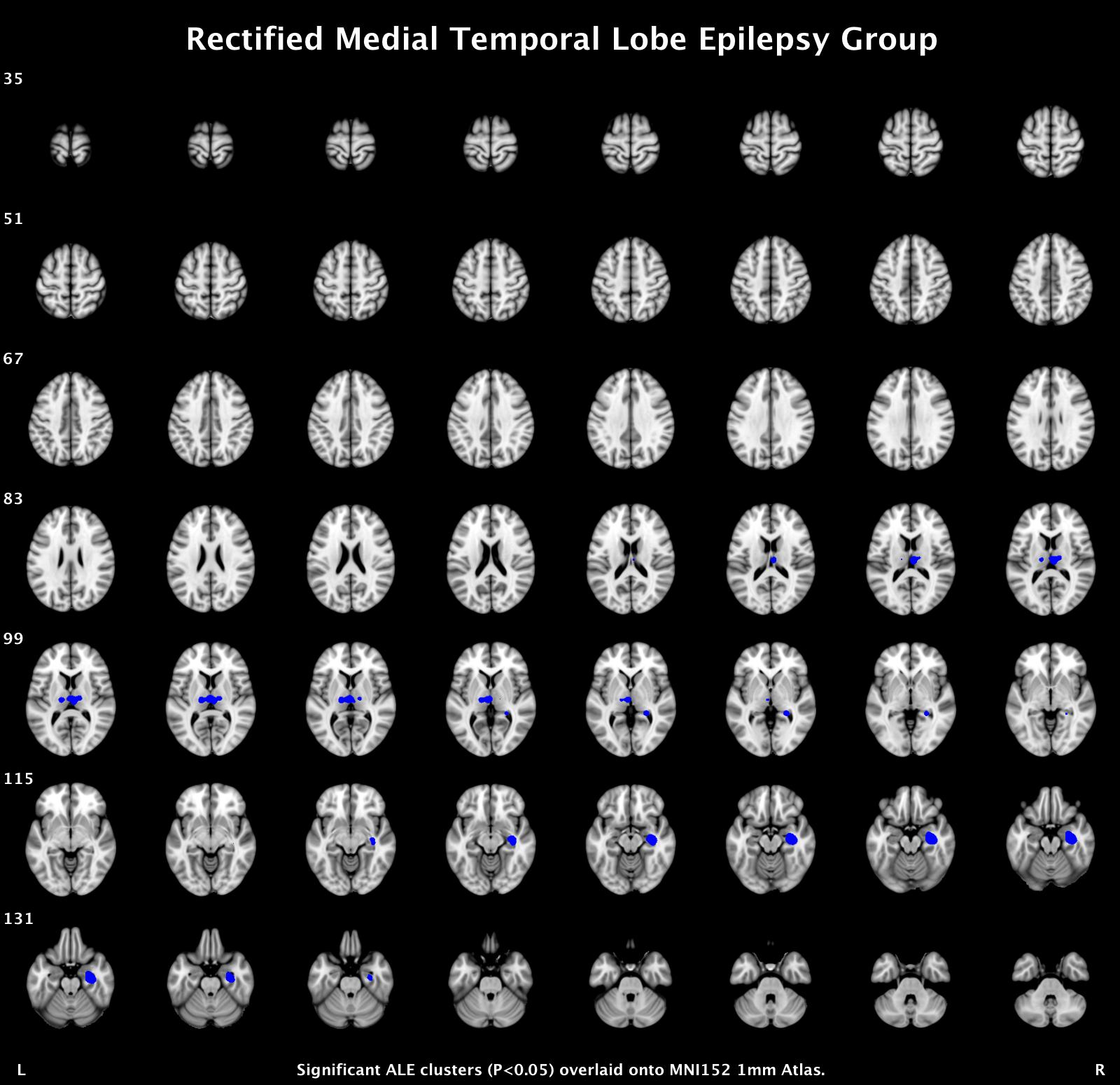


**SupportingFigure 2:**


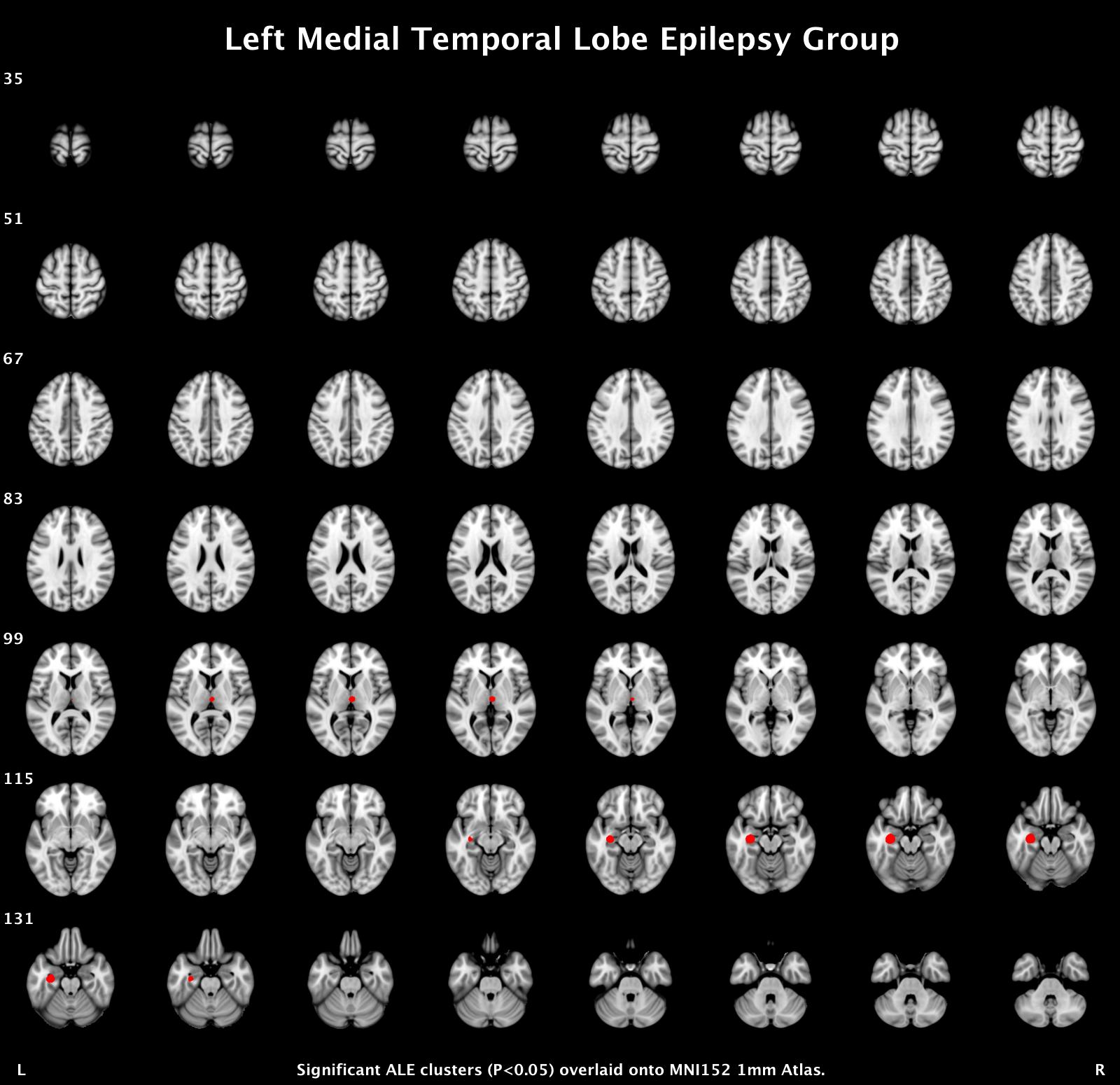


**Supporting Figure 3:**


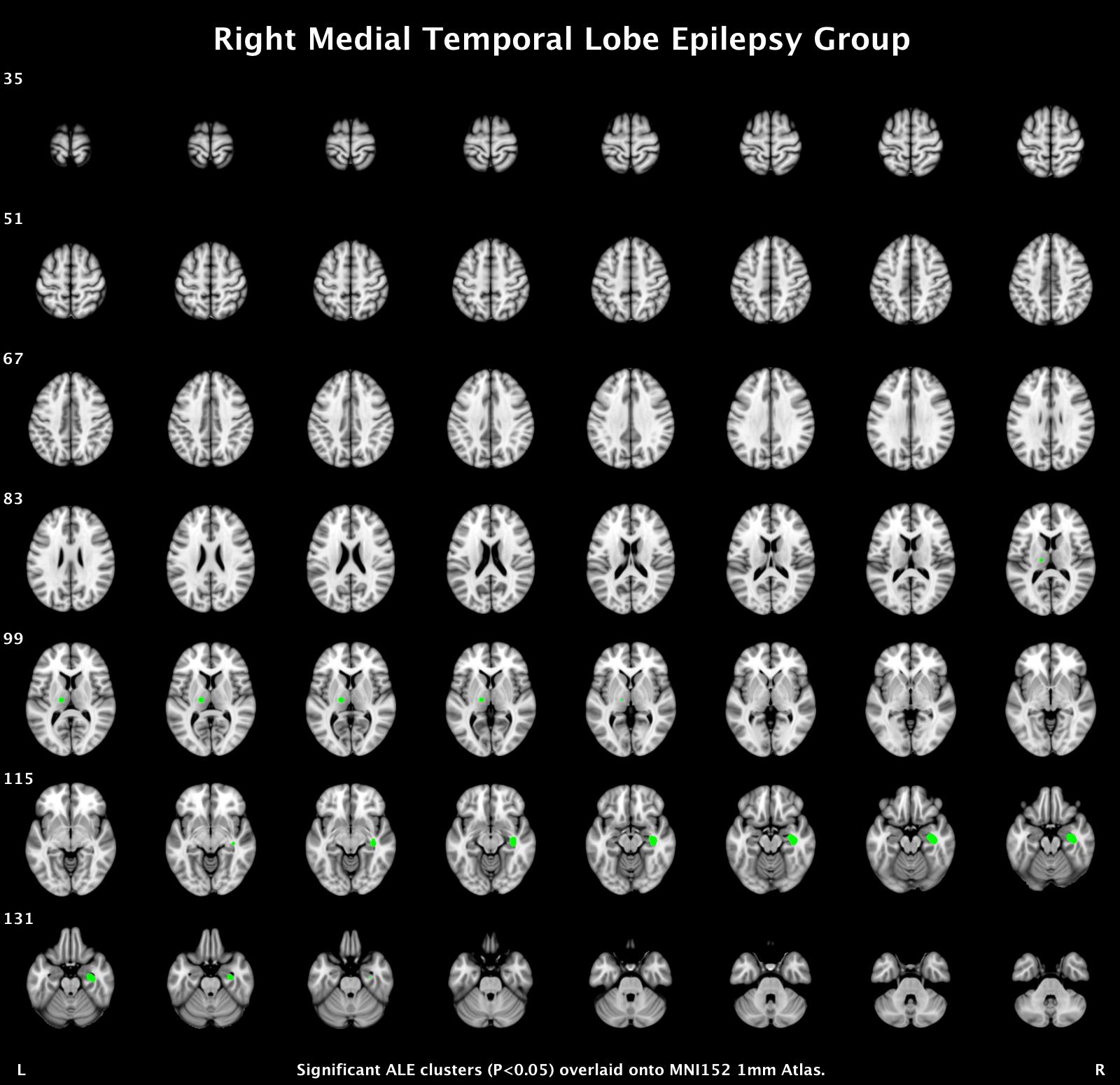

Supplement: Supplementary file 1 — Supplementary materials. [file mmc1.docx]
